# Trade-offs of lipid remodeling in a marine predator–prey interaction in response to phosphorus limitation

**DOI:** 10.1073/pnas.2203057119

**Published:** 2022-08-29

**Authors:** Richard Guillonneau, Andrew R. J. Murphy, Zhao-Jie Teng, Peng Wang, Yu-Zhong Zhang, David J. Scanlan, Yin Chen

**Affiliations:** ^a^School of Life Sciences, University of Warwick, Coventry CV4 7AL, United Kingdom;; ^b^College of Marine Life Sciences, Ocean University of China, Qingdao 266005, China;; ^c^Frontiers Science Center for Deep Ocean Multispheres and Earth System, Ocean University of China, Qingdao 266005, China;; ^d^State Key Laboratory of Microbial Technology, Marine Biotechnology Research Center, Shandong University, Qingdao 266237, China

**Keywords:** predation, marine ciliate, roseobacters, lipid remodeling, phosphorus limitation

## Abstract

Phosphorus (P) is a key nutrient limiting bacterial growth and primary production in the oceans. Unsurprisingly, marine microbes have evolved sophisticated strategies to adapt to P limitation, one of which involves the remodeling of membrane lipids by replacing phospholipids with non-P-containing surrogate lipids. This strategy is adopted by both cosmopolitan marine phytoplankton and heterotrophic bacteria and serves to reduce the cellular P quota. However, little, if anything, is known of the biological consequences of lipid remodeling. Here, using the marine bacterium *Phaeobacter* sp. MED193 and the ciliate *Uronema marinum* as a model, we sought to assess the effect of remodeling on bacteria–protist interactions. We discovered an important trade-off between either escape from ingestion or resistance to digestion. Thus, *Phaeobacter* grown under P-replete conditions was readily ingested by *Uronema*, but not easily digested, supporting only limited predator growth. In contrast, following membrane lipid remodeling in response to P depletion, *Phaeobacter* was less likely to be captured by *Uronema*, thanks to the reduced expression of mannosylated glycoconjugates. However, once ingested, membrane-remodeled cells were unable to prevent phagosome acidification, became more susceptible to digestion, and, as such, allowed rapid growth of the ciliate predator. This trade-off between adapting to a P-limited environment and susceptibility to protist grazing suggests the more efficient removal of low-P prey that potentially has important implications for the functioning of the marine microbial food web in terms of trophic energy transfer and nutrient export efficiency.

## Significance

Microbial growth is often limited by key nutrients like phosphorus (P) across the global ocean. A major response to P limitation is the replacement of membrane phospholipids with non-P lipids to reduce their cellular P quota. However, the biological “costs” of lipid remodeling are largely unknown. Here, we uncover a predator–prey interaction trade-off whereby a lipid-remodeled bacterial prey cell becomes more susceptible to digestion by a protozoan predator facilitating its rapid growth. Thus, we highlight a complex interplay between adaptation to the abiotic environment and consequences for biotic interactions (grazing), which may have important implications for the stability and structuring of microbial communities and the performance of the marine food web.

Bacteria play fundamental roles in the functioning of the ocean ecosystem, being at the base of marine food webs and via mediating key biogeochemical cycles (1–3). One of the key nutrients that constrains microbial growth in the oceans is phosphorus (P) (4), with, e.g., the North Atlantic Ocean and the Mediterranean Sea being well-known P-limited environments (5–8). P is an essential element for all cells, forming the backbone of nucleic acids, adenosine triphosphate, and membrane phospholipids. P limitation can also affect the complex interplay between heterotrophic bacteria and phytoplankton, thus influencing trophic interactions and the cycling of organic matter (9, 10). Global change is expected to exacerbate P limitation in the surface ocean due to water-column stratification accelerated by global warming (11). As such, it is not surprising that marine microbes have evolved sophisticated mechanisms to cope with P limitation, with one such strategy being the substitution of membrane phospholipids by non-P-containing surrogate lipids. Indeed, during P deficiency, both photosynthetic cyanobacteria and algae (12), as well as heterotrophic bacteria of the SAR11 and roseobacter clades, are capable of reducing their consumption of P by substituting membrane phospholipids with non-P-containing versions (13–15). In heterotrophic bacteria, this so-called lipid-remodeling pathway is mediated by the *plcP*-encoded phospholipase C enzyme (16, 17). Previous metagenomics analysis estimates that up to one-quarter of bacteria in surface-ocean assemblages possess the *plcP* gene (14, 18). Indeed, its abundance appears to be a reliable biomarker for phosphate availability in marine surface waters (6). We and others have also shown that PlcP-mediated lipid remodeling occurs naturally in surface seawater microbial assemblages (14, 18). Thus, non-P lipids, primarily glycolipids and betaine lipids, have been shown to account for >70% of the polar lipids in summer, when P is severely limited, but only ∼30% in the autumn, when P limitation is alleviated (14).

It has become increasingly evident that the ability to perform lipid remodeling offers a competitive advantage to bacteria, saving up to 50 to 86% of cellular P demand (13, 14), although genome streamlining may also play a role in this respect (19, 20). However, it remains unknown whether such a drastic change in membrane composition has unforeseen consequences for other aspects of bacterial physiology, particularly how these organisms interact with their biotic environment. In general terms, the abundance of marine microbes is governed by the interplay between abiotic (light and nutrients including P) and biotic (viral lysis and protist grazing) factors (21), which dictates the overall balance between bacterial growth and mortality, and ultimately their population size in the global ocean. While much is known of the consequences of biotic controls on the mortality of marine microbes (22, 23), with estimates suggesting that almost all the daily bacterial production can be removed by viral lysis and protozoan grazing (21, 24), much less is known about how these biological controls are affected by the abiotic environment. This is especially the case when considering the interplay between cosmopolitan marine bacteria and protist predators and, particularly, how adaptation to the abiotic nutrient environment affects predator–prey interactions (25, 26). Protists are one of the most diverse and abundant groups of marine microbes (27–29), among which the ciliates are an important taxon with a global distribution (30) capable of exerting considerable selective pressure on bacterioplankton dynamics through grazing. Indeed, ciliates can directly influence the structure and abundance of dominant marine heterotrophic bacteria, such as the marine roseobacter clade, which are key players in key biogeochemical cycles via their formation of climate-active trace gases, such as dimethylsulfide and methylamines (31, 32).

Here, we specifically set out to investigate the interplay between abiotic and biotic factors in controlling interactions between marine microbes. Using a marine ciliate, *Uronema marinum*, and a marine roseobacter clade bacterium (*Phaeobacter* sp. MED193) as a model, we examine how the dynamics of prey–predator interactions are influenced by P availability. We reveal an important trade-off for a lipid-remodeled prey, which is more susceptible to digestion by a ciliate predator compared to its unmodeled counterpart, despite the latter being more readily ingested by the predator.

## Results

### Establishing a Model System to Investigate the Impact of Predator–Prey Interactions under P Limitation.

**Fig. 1.**
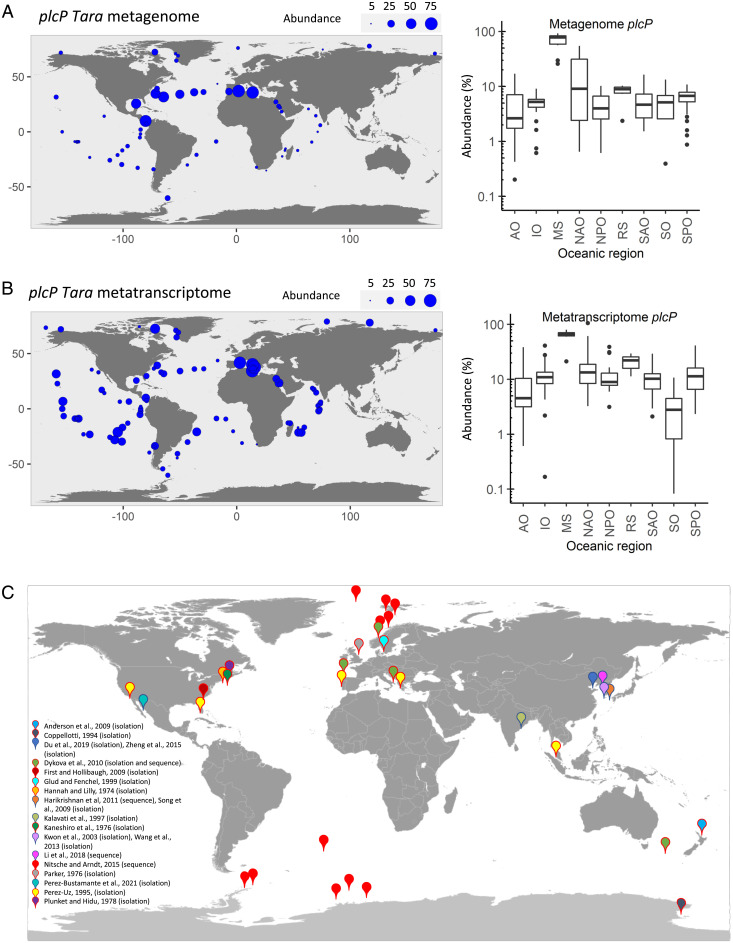
Global prevalence and expression of the *plcP* gene and geographic distribution of *Uronema* sp. in marine systems. The distribution of the *plcP* gene in the global ocean (surface waters only) using the Tara Oceans dataset is shown. The area of each bubble represents the normalized *plcP* gene (*A*) or transcripts (*B*) abundance as the percentage of the median of 10 prokaryotic single-copy marker genes/transcripts at each sampling site. The OGA database was searched by using *plcP* of *Phaeobacter* sp. MED193 with an e-value cutoff of e^−40^. AO, Arctic Ocean; IO, Indian Ocean; MS, Mediterranean Sea; NAO, North Atlantic Ocean; NPO, North Pacific Ocean; RS, Red Sea; SAO, South Atlantic Ocean; SO, Southern Ocean; SPO, South Pacific Ocean. (*C*) Geographic distribution based on culture isolation and molecular detection of *U. marinum* strains around the world from various representative studies (*SI Appendix*, Table S1).

We first examined the active prevalence of PlcP-mediated lipid remodeling across the global ocean using Tara Oceans metatranscriptome datasets. In agreement with previous analyses of metagenomics datasets, which showed the high abundance of the *plcP* gene in the North Atlantic Ocean, Red Sea, and Mediterranean Sea (Fig. 1*A*) (6, 13, 14), on average, ∼20% of surface marine bacteria have *plcP*. In the Mediterranean Sea, transcription of *plcP* was >10-fold higher (Fig. 1*B*) than other ocean regions that are not typically P-limited, such as the Southern Ocean (4), highlighting the importance of lipid remodeling as a key process enabling the proliferation of marine microbes inhabiting P-deplete oceanic waters. Given the high expression of *plcP* in the Mediterranean Sea, Red Sea, and North Atlantic Ocean, we used a model marine roseobacter clade bacterium, *Phaeobacter* sp. MED193, originally isolated from these Mediterranean waters (33), as prey and that is already known to undergo lipid remodeling during P-deplete growth (12). Similarly, as a model predator, we used the marine ciliate *U. marinum* (hereafter *Uronema*), which has been readily isolated from the Mediterranean Sea (34, 35), but is globally distributed (Fig. 1*C*), being widely reported in numerous studies (*SI Appendix*, Table S1).

### Lipid-Remodeled *Phaeobacter* sp. MED193 Prey Is Less Readily Ingested by the Ciliate Predator.

**Fig. 2.**
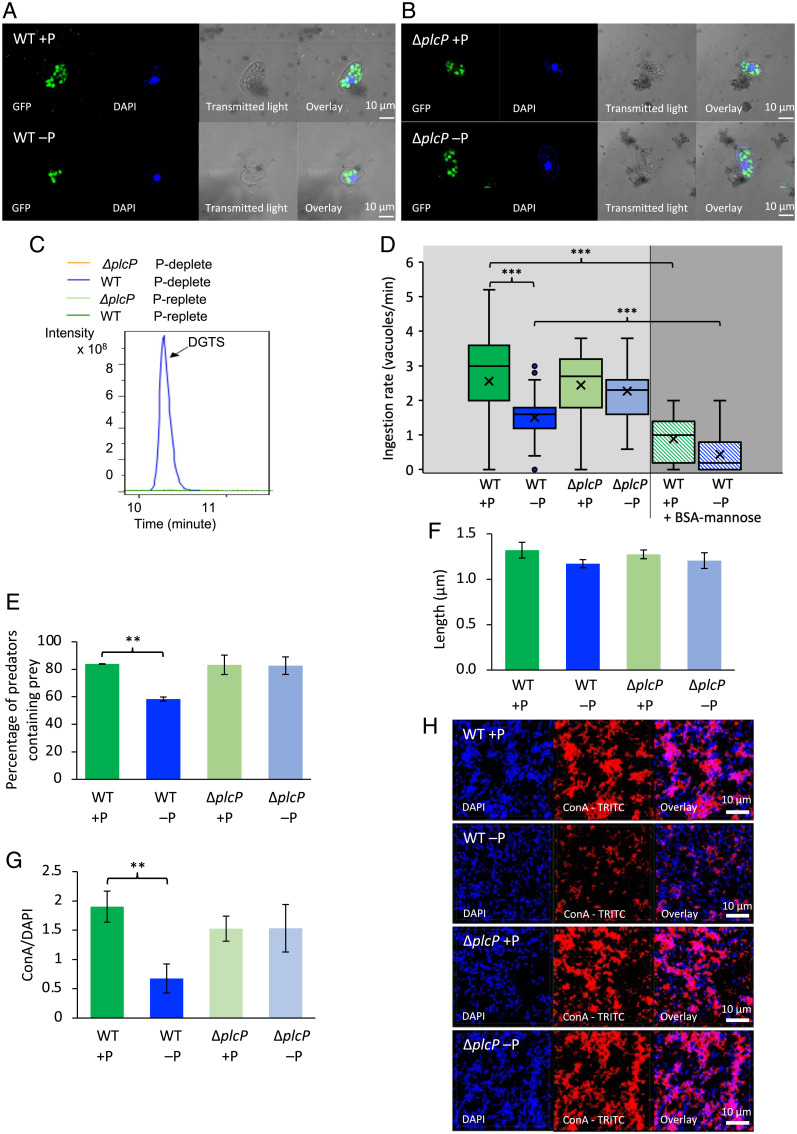
Lipid remodeling facilitates prey escape from ingestion by the marine ciliate *U. marinum*. (*A* and *B*) CLSM images showing GFP-labeled WT prey (*A*) or the Δ*plcP* mutant (*B*) inside the predator. WT and mutant prey cells were grown under either P-replete or P-deplete conditions. Formation of the surrogate lipid DGTS is a hallmark event for lipid remodeling, which was observed only in the WT prey cultivated in P-deplete conditions (*C*). (*D* and *E*) Both ingestion rate (*D*) and the percentage of prey inside the protist (*E*) revealed that lipid-remodeled prey was less likely ingested by the ciliate. These experiments were carried out in three biological replicates, and at least 100 ciliates were counted for each replicate. (*F*) Measurement of the cell length of WT and Δ*plcP* mutant cells. The measurements were carried out by using MicrobeJ software with at least 300 cells for each of the three replicates. (*G* and *H*) CLSM images (*H*) and biovolume quantification (*G*) showing detection of mannose-containing glycoconjugates by TRITC (red)-labeled ConA lectin (ConA-TRITC). The ratio was expressed as the biovolume of ConA-stained glycoconjugates to the bacterial biovolume (DAPI; blue). Values are the mean of three replicates; error bars represent SDs, and asterisks indicate significant difference. **P < 0.01; ***P < 0.001.

Because the bacterial cell membrane is one of the first points of contact during predation by phagotrophic cells, we first examined predator–prey interactions during short-term feeding experiments (Fig. 2). Confocal microscopy showed that when *Uronema* fed on wild-type (WT) *Phaeobacter* prey labeled with green fluorescent protein (GFP) to aid visualization within the ciliate and cultivated in P-replete artificial seawater (ASW), a higher number of prey were observed inside the predator than the same prey cultured under P-deplete conditions (Fig. 2*A*). This suggested that lipid-remodeled prey were less likely to be captured by the predator and that substitution of membrane phospholipids by non-P-containing lipids (i.e., diacylglyceryltrimethylhomoserine [DGTS] in *Phaeobacter* sp. MED193) facilitated prey escape from predation. Given that a *Phaeobacter* sp. MED193 Δ*plcP* mutant is unable to perform lipid remodeling and, hence, cannot produce the surrogate lipid DGTS (14), we investigated whether predator–prey interactions were affected in the Δ*plcP* mutant. We found no difference in ingestion of the Δ*plcP* mutant prey—i.e., no obvious differences in prey numbers inside the protist—regardless of whether the mutant prey was cultured in P-replete or P-deplete conditions (Fig. 2*B*). Lipidomics analysis confirmed that lipid remodeling (evidenced by formation of DGTS) only occurred in WT prey cultivated under P-deplete conditions, while the Δ*plcP* mutant did not produce DGTS under either P-deplete or P-replete growth conditions (Fig. 2*C* and *SI Appendix*, Fig. S1). Together, these results suggest that the ciliate is capable of differentiating prey types, depending on their ability to perform lipid remodeling and synthesis of non-P surrogate membrane lipids.

To more quantitatively assess this difference in prey preference, we analyzed the ingestion rate—i.e., the number of bacteria ingested per minute—using either WT or Δ*plcP* mutant prey. When consuming WT GFP-labeled prey cultivated under P-replete conditions, *Uronema* was able to ingest 2.55 ± 1.45 bacteria/min, while for the same prey grown under P-deplete conditions, *Uronema* only ingested 1.51 ± 0.66 bacteria/min (Fig. 2*D*). In contrast, there was no difference in ingestion rate with Δ*plcP* mutant prey grown under P-replete and P-deplete conditions (Fig. 2*D*). We also analyzed the percentage of protozoa containing prey using the WT or Δ*plcP* mutant (Fig. 2*E*). Thus, after 5 min of interaction, 84 ± 0.2% of protozoa contained prey when interacted with WT grown under P-replete conditions, but the number reduced to 58 ± 1.5% when WT prey was cultivated under P-deplete conditions. Conversely, no difference in the percentage of protozoa containing prey was observed when the Δ*plcP* mutant was grown under P-replete/-deplete conditions (Fig. 2*E*). Together, these results support the notion that P-stress-induced membrane lipid remodeling facilitates prey escape from ciliate predation.

### Escape from Ciliate Ingestion Involves Mannosylated Glycoconjugates, but Not Size Selection.

We then set out to investigate the underlying mechanism for the apparent predator avoidance of lipid-remodeled prey. Since protists are well known for selecting prey according to size (36), we first examined whether there was a difference in size between the WT and Δ*plcP* mutant grown under P-replete and P-deplete conditions. However, no obvious size differences were observed (Fig. 2*F*). In addition to size, masking the cell surface is another common strategy adopted by prey to avoid ingestion by protists (36). We therefore investigated whether the formation of surrogate membrane lipids under P-deplete conditions affected prey capture by predator phagocytic receptors, among which the mannose receptor is strikingly conserved across the eukaryotic domain (37) and known to be used as a feeding receptor for recognizing prey (38). We studied whether there was a noticeable difference in mannose-decorated glycoconjugates in WT and *plcP* mutant prey grown under P-replete and P-deplete conditions using a mannose-binding lectin (Concanavalin A [ConA]) labeled with rhodamine. When WT prey was grown in P-replete medium, substantially more rhodamine-labeling was detected by confocal microscopy (Fig. 2 *G* and *H*), compared to the same prey grown under P-deplete conditions, where the rhodamine signal was significantly reduced (Fig. 2 *G* and *H*). This difference in rhodamine labeling was not observed with Δ*plcP* mutant prey, which is unable to perform lipid remodeling (Fig. 2 *G* and *H*). These results suggest that the formation of surrogate lipids in response to P limitation reduces the level of mannose-containing glycoconjugates on the prey cell surface.

To further support the involvement of mannose receptors in prey capture by *Uronema*, a short-term ingestion experiment was performed by using ciliates that had been preincubated with mannosylated bovine serum albumin (BSA-mannose) as a decoy. After incubation of the predator with BSA-mannose, a significantly lower rate of bacteria was ingested by the protozoa, corresponding to 0.89 ± 0.71 bacteria/min and 0.45 ± 0.57 bacteria/min for the WT grown under P-replete or P-deplete medium, respectively (Fig. 2*D*). Together, these experiments suggest that lipid remodeling in response to P limitation affects mannosylated macromolecules that are involved in prey–predator recognition.

### The Ciliate Predator Is Capable of Selective Grazing with a Preference for Prey Incapable of Lipid Remodeling.

Since prey carrying out lipid remodeling reduce the expression of mannose-containing glycoconjugates, which, in turn, reduces ingestion by the predator, we hypothesized that this would result in selective grazing by the predator in a mixed-prey community. To test this idea, we carried out three types of predation experiments using 1) WT prey grown under P-replete conditions mixed with WT prey grown under P-deplete conditions; 2) WT prey and Δ*plcP* mutant prey both cultivated under P-replete conditions; and 3) WT prey and Δ*plcP* mutant prey both cultivated under P-deplete conditions. To facilitate microscopy of the prey inside the ciliate, WT and Δ*plcP* mutant prey were labeled with GFP and mCherry, depending on the growth condition, for subsequent confocal imaging.

**Fig. 3.**
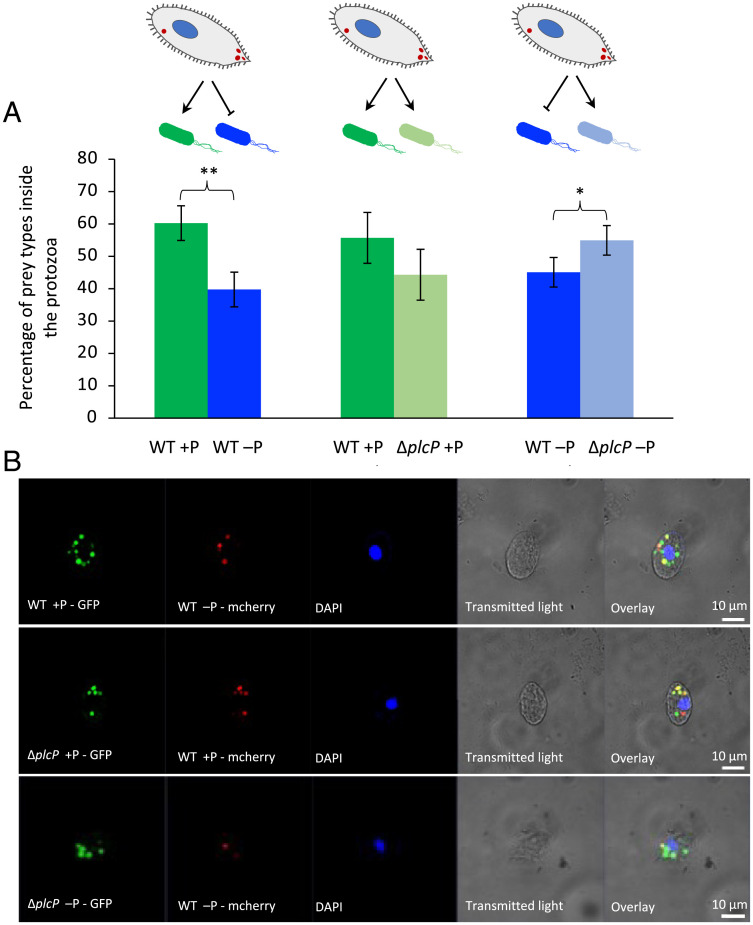
The marine ciliate *U. marinum* selectively ingests lipid-unmodeled prey (*A*) Percentage of phagosomes containing WT prey grown under P-replete or P-deplete conditions (*Left*), WT or Δ*plcP* mutant prey grown under P-replete conditions (*Center*), or WT and Δ*plcP* mutant prey grown under P-deplete conditions (*Right*). Results were obtained from three independent biological replicates counting food vacuoles from *n* = 100 ciliates. Values are the mean of three replicates; error bars represent SDs, and asterisks indicate significant difference. *P < 0.05; **P < 0.01. (*B*) CLSM images of prey inside the predator. (*B*, *Top*) WT grown under P-replete conditions (labeled with GFP) and WT grown under P-deplete conditions (labeled with mCherry). (*B*, *Middl*e) WT (labeled with mCherry) and the Δ*plcP* mutant (labeled with GFP) grown under P-replete conditions. (*B*, *Bottom*) WT (labeled with mCherry) and the Δ*plcP* mutant (labeled with GFP) grown under P-deplete conditions.

When the ciliate had the choice to feed on WT prey that was previously cultivated in a medium either replete or deplete in P (the latter condition causing lipid remodeling), the ciliate preferentially ingested prey that had not undergone lipid remodeling. Thus, after 5 min of interaction, there were 60.3 ± 5.4% P-replete prey, but only 39.7 ± 5.4% P-deplete prey (Fig. 3 *A* and *B*, *Top*). In contrast, when the ciliate had the choice between WT and Δ*plcP* mutant prey that had both been grown under P-replete conditions, *Uronema* showed no significant prey preference (*P* > 0.05; Fig. 3 *A* and *B*, *Middle*), consistent with no lipid remodeling occurring under these conditions. Finally, *Uronema* given the choice of WT and Δ*plcP* mutant prey both grown in P-deplete medium (and, hence, with only WT prey having undergone lipid remodeling), the ciliate displayed a clear preference for the Δ*plcP* mutant (*P* < 0.05; Fig. 3 *A* and *B*, *Bottom*). Together, these selective grazing experiments using a mixed-prey community support the finding that a lipid-remodeled prey has a selective advantage over its nonmodeled counterpart by reducing its susceptibility to protist ingestion.

### A Lipid-Remodeled Prey Supports Better Growth of the Ciliate.

**Fig. 4.**
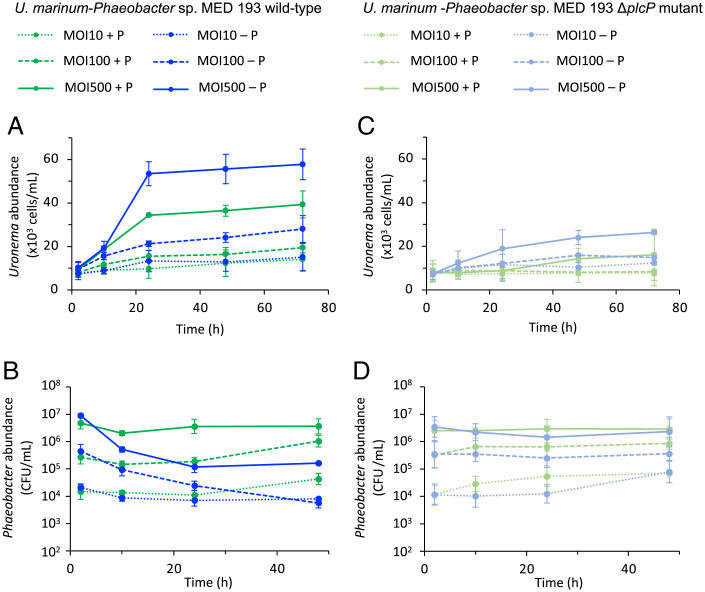
Lipid-remodeled *Phaeobacter* sp. MED193 prey promotes better growth of *Uronema*. The abundance of *U. marinum* (*A* and *C*), *Phaeobacter* sp. MED193 WT (*B*), and Δ*plcP* mutant (*D*) during interactions in P-replete (green) or P-deplete (blue) medium at MOI 10 (dotted lines), MOI 100 (dashed lines), and MOI 500 (solid lines). *Uronema* cells were counted by using a Malassez counting chamber over a 72-h interaction period, and *Phaeobacter* WT and Δ*plcP* mutant cells were counted over a 48-h period of interaction with *Uronema*. Measurements were made by using three biological replicates, each with three technical replicates, and error bars represent SDs.

Having established that lipid remodeling facilitates the escape of prey from ingestion by the ciliate predator, we next examined the fate of prey in a long-term interaction experiment performed up to 72 h. Interestingly, WT prey capable of lipid remodeling supported better growth of the ciliate at all three multiplicities-of-infection (MOIs) tested (Fig. 4*A*). Thus, after 24 h of interaction at a MOI of 500, *Uronema* cell densities reached 53,480 ± 5,517 cells/mL when fed on P-deplete prey compared to 34,370 cells/mL when fed on P-replete prey (Fig. 4*A*). Concomitantly, a sharp decrease in prey numbers occurred between 3 h and 24 h of interaction (Fig. 4*B*). The same trends were seen with MOIs of 100 and 10. Growth of the predator at 24-h interaction was much reduced when consuming P-deplete Δ*plcP* mutant prey (18,889 ± 8,694 cells/mL) that is unable to perform lipid remodeling (Fig. 4*C*), and there was little difference in *Uronema* growth when fed on P-replete/-deplete Δ*plcP* mutant-grown prey. Consequently, Δ*plcP* mutant prey abundance decreased only slightly across the experiment (Fig. 4*D*). Control experiments demonstrated that medium alone did not support *Uronema* or *Phaeobacter* growth and that the addition of Triton X-100 to release bacteria from protozoa had no impact on prey viability (*SI Appendix*, Fig. S2). This significantly better *Uronema* growth when fed WT prey that has undergone lipid remodeling (Fig. 4*A*) suggests that lipid-remodeled prey cells are more easily digested by the ciliate once ingested.

### Lipid-Remodeled Prey Is More Susceptible to Digestion.

**Fig. 5.**
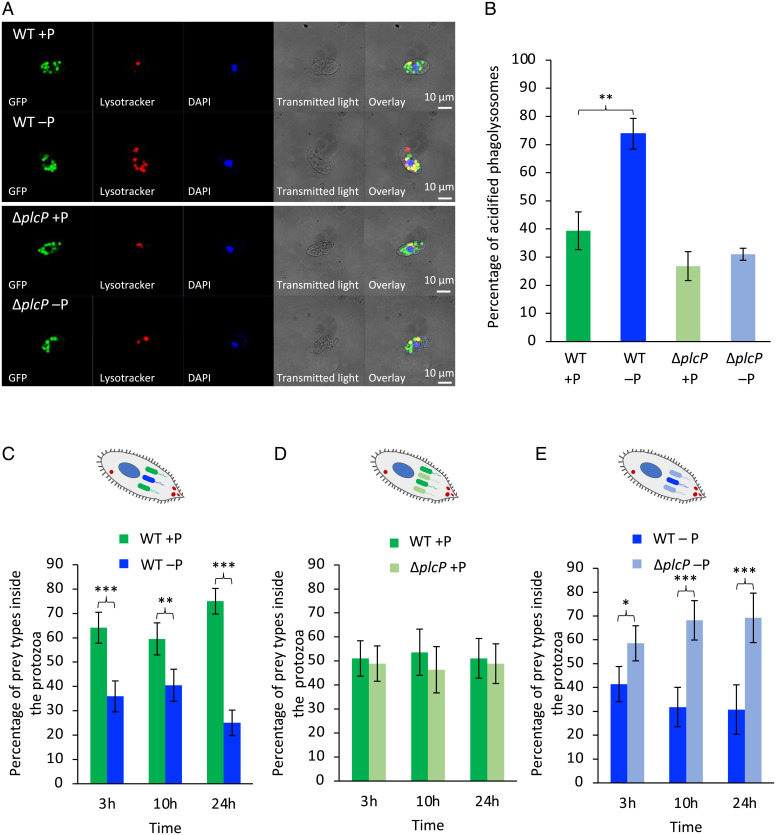
A lipid-remodeled prey is more susceptible to ciliate digestion inside the phagolysosome. (*A*) CLSM images showing GFP-labeled WT prey (*Upper*) or Δ*plcP* mutant prey (*Lower*) and acidification of the phagolysosomes (Lysotracker, red) during predation by *Uronema*. (*B*) The percentage of acidified phagolysosomes containing WT prey or Δ*plcP* mutant prey inside the predator. Values are the mean of three replicates; error bars represent SDs, and asterisks indicate significant difference. **P < 0.01. (*C*) The percentage of phagolysosomes containing WT prey that had been grown under P-replete or P-deplete conditions. (*D*) The percentage of phagolysosomes containing either WT or Δ*plcP* mutant prey grown in P-replete medium. (*E*) The percentage of phagolysosomes containing WT or Δ*plcP* mutant prey grown in P-deplete medium. For *C*–*E*, results were obtained from three independent biological replicates, from which vacuoles from *n* = 100 ciliates per replicate were counted. Values are the mean of three replicates; error bars represent SDs, and asterisks indicate significant difference. *P < 0.05; **P < 0.01; ***P < 0.001.

In order to understand the fate of lipid-remodeled prey inside the ciliate, colocalization studies were performed by tracking phagolysosome acidification after prey engulfment using the LysoTracker Red DND-99 stain, which specifically stains acidic organelles in live cells. Confocal imaging showed intensive acidification of prey-containing vacuoles after 3-h interaction between *Uronema* and WT prey grown under P-deplete conditions, whereas WT prey cultivated under P-replete conditions markedly reduced acidification of the vacuoles (Fig. 5*A*). The Δ*plcP* mutant was also capable of largely preventing vacuole acidification, regardless of whether it was cultivated in P-replete or P-deplete conditions. Thus, a quantitative analysis showed that only 39.4 ± 6.7% of bacteria-containing vacuoles were acidified when *Uronema* was fed P-replete prey, compared to 73.8 ± 5.5% bacteria-containing vacuoles being acidified when fed P-deplete WT prey (Fig. 5*B*). Corresponding data for Δ*plcP* mutant prey grown under P-replete/P-deplete conditions was 26.8 ± 5.1% and 31.0 ± 2.1%, respectively. Taken together, these results suggest that, once ingested, lipid-remodeled prey is unable to prevent acidification of the phagosome and, as such, is better digested by the ciliate, therefore supporting higher *Uronema* cell abundances.

Such an idea is further supported by analysis of food-containing vacuoles in mixed predator–prey interaction experiments. After 3-h, 10-h, and 24-h interaction between *Uronema* and *Phaeobacter*, when *Uronema* was fed WT and Δ*plcP* mutant prey grown under P-replete conditions, there was no major change in the percentages of the two prey types inside the predator (Fig. 5*D*). However, when WT lipid-remodeled prey was mixed with either WT or Δ*plcP* mutant unmodeled prey, the former was selectively digested (Fig. 5 *C* and *E*). Together, these data suggest that prey that has undergone lipid remodeling is less able to inhibit phagolysosomal acidification after engulfment and, as such, is more easily digested, supporting better growth of the predator. Indeed, assessment of *Phaeobacter* survival in medium mimicking acidification (pH 6) and oxidative stress (the presence of hydrogen peroxide) conditions inside a phagolysosome showed that the WT lipid-remodeled prey survived poorly at lower pH or in the presence of H_2_O_2_ compared to its unmodeled counterpart (*SI Appendix*, Fig. S3).

## Discussion

**Fig. 6.**
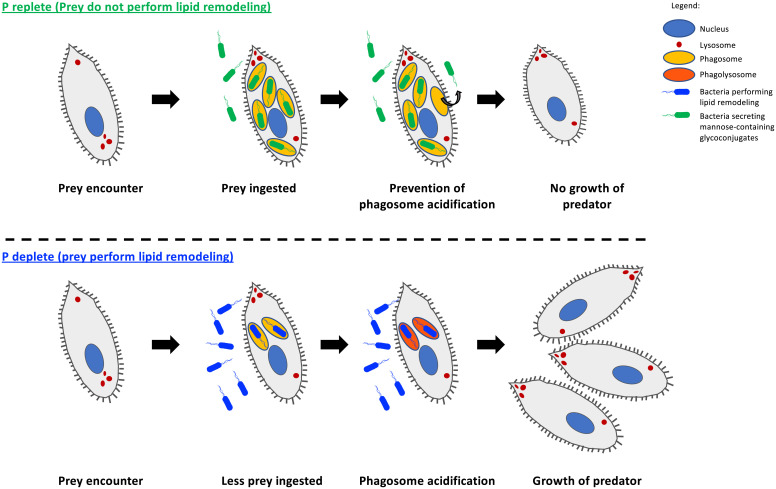
Proposed trade-off mechanism due to lipid remodeling of *Phaeobacter* sp. MED 193 when interacting with the predator *U. marinum. Phaeobacter* sp. MED193 grown in a P-deplete environment induces lipid remodeling in order to reduce its P quota and release phosphate for use in other major cellular processes. A lipid-remodeled cell has a reduced level of mannose-containing macromolecules, making prey capture by *U. marinum* problematic. However, conversely, a lipid-remodeled prey is unable to prevent phagolysosomal acidification, making it more susceptible to digestion by the ciliate, which, in turn, facilitates better ciliate growth.

The low availability of key nutrients like P in marine surface waters represents a grand challenge for microbes, particularly those inhabiting oligotrophic gyres. Although lipid remodeling enables these microbes to survive better in these potentially P-limited environments, as well as facilitating greater avoidance of ingestion by ciliate grazers, once ingested, these lipid-remodeled cells are unable to survive phagolysosomal digestion (Fig. 6). Therefore, these microbes face an unsolvable dilemma. On the one hand, lipid remodeling helps prey escape ingestion by the ciliate, but on the other, prey is more susceptible to insult from the harmful acid and oxidative stress conditions resulting from lysosome fusion and phagosome acidification. We should add, though, that such a dilemma (between escape from ingestion and resistance to digestion) is not the only trade-off that microbes face due to grazing pressure. For example, it is well known that bacteria can form biofilms or clumping (39, 40) to avoid capture by protists at the expense of growth, with such morphological changes affecting cell-surface area-to-volume ratios, which negatively impact on nutrient uptake for optimum growth. Similarly, unicellular algae with larger cell sizes are poorly grazed, but have low ammonium uptake rates and grow poorly (41). Equally, *Pseudomonas aeruginosa* isolated from long-term chronically infected patients are less resistant to protist grazing (42). Thus, it is clear that adaptation to a specific niche can come with consequences to an organism’s viability, although it remains to be seen what other trade-offs in predator–prey interactions exist following adaptation of cosmopolitan marine microbes to P limitation.

To capture prey, phagotrophic cells must recognize certain bacterial structures to avoid self-predation or engulfment of inorganic matter (43). One of the most evolutionally conserved structures involved in prey recognition by phagotrophic cells is mannose-binding lectins (44), which are found not only in mammalian macrophages, but also protists, such as dinoflagellates and ciliates (37). Interestingly, we observed that lipid remodeling induced by prey P limitation impaired decoration of prey cells by mannose-containing glycoconjugates and subsequent ingestion by the ciliate grazer (Fig. 2 *G* and *H*), although the identities of these cell-surface-mannosylated glycoconjugates remain to be established. Such an interplay of abiotic control of prey ingestion may be more common than previously expected. For example, it has been noted that a nitrogen-deficient culture of the microalgae *Isochrysis galbana* was also rich in cell-surface mannose (45, 46), while phosphate limitation appears to play a role in the digestion of green algae by zooplankton (47). Moreover, members of the highly abundant SAR11 clade have a much less hydrophobic cell surface than other planktonic bacteria, facilitating filtration evasion by slipping through the mucous nets of marine tunicates (48). This reiterates the importance and complexity of the prey cell surface in predator–prey interactions.

Surprisingly, enhanced ingestion of prey did not translate into better growth of the ciliate predator. While we would have expected better *Uronema* growth when grazing on WT prey that had previously grown in P-replete medium, due to its higher nutritional quality (49), we observed the opposite (Fig. 4). Thus, 24 h postinteraction with WT prey cultivated in P-replete medium *Uronema* reached a concentration 35% lower (at a MOI of 500) compared to the same prey cultivated in P-deplete medium. Concomitantly, nearly 99% of the prey cultivated in P-replete medium were consumed (Fig. 4*B*). It thus appears that lipid-unmodeled prey (i.e., WT prey grown in P-replete conditions or the Δ*plcP* deletion mutant grown under either P-replete or P-deplete conditions—since this mutant is unable to remodel its lipids) is capable of preventing digestion by inhibiting the acidification of the food-containing vacuole. This conclusion is supported not only by colocalizing fluorescence-labeled prey cells with LysoTracker (Fig. 5 *A* and *B*), but also by the evolution of intracellular survival in mixed-prey grazing experiments following the preculturing of prey under P-replete or P-deplete growth conditions (Fig. 5 *C*–*E*). Interestingly, in vitro experiments mimicking phagolysosome acidification and oxidative stress also demonstrate that lipid-remodeled prey are significantly more sensitive to killing by low pH and oxidative stress (*SI Appendix*, Fig. 2). Although it is unclear how membrane lipid remodeling prevents acidification of the food-containing vacuoles in this ciliate, it is possible that some type of bacterial effector is released by the prey in response to mannose-binding lectin-dependent phagocytosis in order to prevent the formation or subsequent acidification of the phagolysosome. In this regard, such a prey–ciliate interaction resembles pathogen clearance by professional human phagocytes, such as macrophages (50). Certainly, marine roseobacters are known to produce toxins (51, 52) that are capable of inhibiting the growth of—or even killing—eukaryotic phytoplankton (52–54). Thus, the role of lipid remodeling in bacteria–eukaryote interactions clearly warrants further investigation.

Overall, our data highlight an avenue in predator–prey interactions—namely, how these interactions can be governed by nutrient availability via the remodeling of membrane lipids that takes place under P-deplete growth conditions (Fig. 6). In the model *Phaeobacter* sp. that we utilize here, remodeling reduces its P consumption by replacing membrane phospholipids (primarily phosphatidylglycerol and phosphatidylethanolamine) with alternative non-P lipids (e.g., DGTS) (14). The fact that this lipid-remodeling process is occurring naturally in P-deplete oceanic waters across not only heterotrophic bacteria (13, 14), but also cyanobacteria and eukaryotic algae (12, 55), is suggestive of a much wider importance of this phenomenon. Extensive lipid remodeling in the natural environment is reiterated not only by metagenomics and transcriptomics data of the *plcP* gene directly involved in remodeling in heterotrophic bacteria at least (Fig. 1*B*), but also by direct analysis of membrane lipids in natural microbial communities (12, 14, 55, 56). However, how general across different taxonomic groups of predator and prey these effects are clearly requires further work. Moreover, given that the effects of remodeling on predator–prey interactions we report here are ultimately controlled by in situ P concentrations (which controls lipid remodeling), then such interaction effects are also likely to be dynamic in their nature, given the often-seasonal nature of P limitation—e.g., in the Mediterranean Sea, PlcP-mediated lipid remodeling occurs across an annual cycle, whereby P limitation intensifies during spring and summer, but starts to become alleviated from September (14, 57). Nonetheless, this work clearly highlights the complex interplay between the abiotic nutrient environment, microbes, and their grazers and how predator–prey dynamics are governed by abiotic control of prey physiology, which has important implications for how we model trophic interactions in marine ecosystem models, particularly in a future scenario where nutrient-deplete gyre regions are set to expand (11).

## Materials and Methods

### Bacteria and Ciliate Strains and Cultivation Conditions.

Bacteria and ciliate strains used in this study are shown in *SI Appendix*, Table S2. *Phaeobacter* sp. MED193 was maintained in Marine Broth (MB) (Difco 2216; BD). A previously constructed *Phaeobacter* sp. MED193 Δ*plcP* mutant, unable to perform lipid remodeling, was also used (14). Bacteria were cultivated in Erlenmeyer flasks containing 40 mL of modified ASW (58, 59), comprising sodium chloride (25 g/L), magnesium chloride hexahydrate (2 g/L), potassium chloride (0.5 g/L), calcium chloride dihydrate (0.5 g/L), magnesium sulfate (1.75 g/L), potassium dihydrogene phosphate (344.6 μM), Hepes (10 mM; pH 8.0), ammonium chloride (0.4078 g/L), and sodium succinate (10 mM). The final pH of the medium was adjusted to 7.6. After autoclaving, the medium was supplemented with 1 mL of trace metal solution (58, 59) and 1 mL of vitamin solution (60). Cultures were incubated at 30 °C with shaking at 180 rpm until stationary phase. To induce lipid remodeling, cells were harvested by centrifugation and washed three times before being resuspended in ASW medium either replete (344.6 μM) or deplete (0 μM) in phosphate and incubated at 30 °C with shaking at 180 rpm for 36 h.

Axenic *U. marinum*, originally isolated from coastal seawater from Qingdao, China (61), was grown in 25-cm^2^ tissue-culture flasks (Corning Falcon) containing 20 mL of Rich’s medium at 22.5 °C. Rich’s medium was modified from the ASW for protozoa (Culture Collection of Algae and Protozoa) and additionally contained 15 g/L protease/peptone (Oxoid), 5 g/L yeast extract (Sigma-Aldrich), 10 g/L Complan (Heinz), 200 mL/L Leibovotz’s L-15 (Fisher), and 0.1 M glucose. Medium was adjusted to pH 7.6 to 7.8 with 2 M sodium hydroxide and autoclaved at 120 °C for 20 min. The axenic nature of the ciliate was routinely monitored by DAPI staining.

### Enumeration of *Uronema*.

*Uronema* growth kinetics were carried out over a time course of 72 h in 96-well microplates. After rapid fixation with 10% Lugol iodine, the concentration of the ciliate suspension was assessed by using a Malassez cell-counting chamber.

### Lipid Analysis.

Bacterial lipids were extracted by using a modified Folch extraction protocol as described (62, 63). Briefly, 1 mL of culture (optical density at a wavelength of 600 nm [OD_600nm_] = 0.5) was collected by centrifugation. Total lipids were then extracted by using methanol–chloroform and dried under nitrogen gas, and the pellet was resuspended in 1 mL of solvent (95% volume [vol]/vol liquid chromatography-mass spectrometry (LC-MS)-grade acetonitrile and 5% 10 mM ammonium acetate, pH 9.2, in water). Bacterial lipids were analyzed by LC-MS using a Dionex 3400RS high-performance LC with a hydrophilic interaction LC ethylene-bridged hybrid amide XP column (2.5 μm, 3.0× 150 mm; Waters) coupled with an amaZon SL ion-trap MS (Bruker) via electrospray ionization in both positive (+ve) and negative (−ve) ionization mode. Data analysis was carried out by using the Bruker Compass software package.

### ConA Staining of Bacteria.

After growth in P-replete or P-deplete ASW medium, bacterial cultures were collected by centrifugation, and the OD_600nm_ was adjusted to 0.1. Then, 1 mL of the different suspensions was inoculated in triplicate into 24-well plates containing a coverslip previously coated with poly-d-lysine (molecular weight 70,000 to 150,000; Sigma-Aldrich). Afterward, the samples were directly fixed by adding formaldehyde at 4% (vol/vol) for 20 min, and a low-speed centrifugation (15 min at 900 × *g*) was used to initiate and increase cell adhesion to the coverslips. Finally, cells were stained with DAPI (5 μg/mL) and ConA TRITC conjugate (20 μg/mL; Thermo Fisher Scientific) (39) and mounted with a drop of Mowiol antifade before observation using a confocal laser scanning microscope (CLSM; Zeiss LSM 880). Bacterial biovolumes and the biovolume of ConA-stained glycoconjugates in CLSM images were determined by using five images for each of the three replicates using COMSTAT software developed in MATLAB R2015a (MathWorks), as described (64). Bacterial cell length was calculated from measurements made by using at least 300 cells for each of the three replicates using MicrobeJ software (65) developed in Fiji 2.1.0/1.53c.

### Prey Labeling Using Fluorescent Proteins.

To visualize prey within protozoa, plasmids pBBR-KanR-p(aphII)-sfGFP and pBBR-KanR-p(aphII)-mCherry were generated that constitutively expressed a GFP or mCherry, respectively. The constitutive promoter of the aminoglycoside phosphotransferase II (*aph*II) was amplified by PCR as described (66), and Gibson assembly was used to drive expression of each fluorescent protein in the broad-host-range cloning vector pBBR1 MCS-2 (67) forming pBBR-GentR-p(aphII)-sfGFP and pBBR-KanR-p(aphII)-mCherry. These plasmids were transformed into *Escherichia coli* S17.1 λ-pir via electroporation and mobilized into WT *Phaeobacter* sp. MED193 and the *plcP* mutant via conjugation (*SI Appendix*, Table S2), using 1/2 yeast extract, tryptone, and sea salts as the medium (DSMZ). Transconjugants were selected by using gentamicin (10 μg/mL) or kanamycin (50 μg/mL) on sea salts minimal medium containing artificial sea salts (30 g/L), sodium phosphate (1 mM), Hepes (10 mM; pH 8.0), FeCl_2_ (5 μM), glucose (3 mM), succinate (5 mM), a mixture of vitamins (68), and glycine betaine (2 mM) as the sole nitrogen source, as described (69).

### Interaction of the Ciliate with a Single Prey.

To initiate physical interaction between the predator and prey and to avoid growth of the prey during interactions, bacteria and protozoa were transferred to ASW medium deplete in either a carbon source (succinate) or phosphate. Protozoan suspensions were inoculated into 96-well plates at 1 × 10^4^ cells/mL. The prey (WT *Phaeobacter* sp. MED193 or the *plcP* mutant) were inoculated at an MOI of 10, 100, or 500 (equivalent to 10, 100, or 500 prey per protist cell, respectively). After 3 h, 10 h, 24 h, and 48 h of contact time at 22.5 °C, ciliates were treated with Triton X-100 (0.05%) for 30 min on ice and mechanically lysed by mixing using a syringe and 25-g needle (BD), as described (39). Bacterial prey numbers were quantified by serial dilution on MB agar plates.

### Interaction of the Ciliate with Two Prey Types.

To initiate interaction of the ciliate with a two-prey mix, prey and ciliate were transferred into ASW medium containing 2.5 mM succinate and 86 µM phosphate (25% of the normal concentration) in order to limit prey growth. The ciliate was inoculated at 1 × 10^4^ cells/mL, whereas bacteria were inoculated at an MOI of 50 for each prey type (i.e., a MOI of 100 for total bacteria) using 24-well plates. After 5 min, 3 h, 10 h, or 24 h of contact time at 22.5 °C, samples were directly fixed by adding formaldehyde at 4% (vol/vol) for 30 min, and then a coverslip previously coated with poly-d-lysine was deposited in each well. Low-speed centrifugation (15 min at 900 × *g*) was used to initiate and increase cell adhesion to the coverslips. Finally, *U. marinum* cells were stained with DAPI and mounted with a drop of Mowiol antifade as described above before observation using CLSM.

### Visualization of Prey Inside *U. marinum* Using CLSM.

Once the prey–ciliate interaction was initiated, subsamples of the ciliates after 5 min, 3 h, 10 h, and 24 h of contact time were stained with DAPI (5 μg/mL) to visualize the ciliate nucleus. After 3 h of contact time, *Uronema* cells were treated with LysoTracker Red DND-99 (Fisher) (70 nM final concentration) for 30 min to stain lysosomal acidic compartments to observe the digestion process. To assess the involvement of mannose receptors in prey capture by *Uronema,* protists were preincubated for 30 min with 20 µM BSA-mannose to mask these receptors, according to Wootton et al. (38), prior to prey interaction. Afterward, samples were directly fixed by adding formaldehyde (4% [vol/vol]) for 30 min. Subsequently, a coverslip previously coated with poly-d-lysine was deposited in each well. Low-speed centrifugation (15 min at 900 × *g*) was used to initiate and increase cell adhesion to the coverslips. Finally, cells were mounted with a drop of Mowiol antifade before observation using CLSM.

### Analysis of *plcP* Transcripts in the *Tara* Oceans Database.

Tara Oceans metagenomes (OM-RGCv2+G) and metatranscriptomes (OM-RGCv2+T) were queried by using *Phaeobacter* sp. MED193 PlcP via the Ocean Gene Atlas (OGA) web portal (18). *plcP* abundance in both metagenomes and metatranscriptomes was obtained by using hmmsearch with an expected threshold of 1e^−40^ normalized to the median abundance of 10 single-copy marker genes (70, 71).

### Statistics.

For each experiment, at least three biological replicates were performed, and ≥100 protist cells per replicate were counted. To test for statistically significant differences (*P* < 0.05) between two conditions, a *t* test was performed, and a one-way ANOVA, including the Bonferroni posttest, was performed to compare more than two conditions. The Wilcoxon–Mann–Whitney *U* test was used to test for statistically significant differences (*P* < 0.05) between the distribution of data obtained between two conditions. These tests were performed by using SPSS 26.0 (IBM).

## Supplementary Material

Supplementary File

## Data Availability

All study data are included in the article and/or *SI Appendix*.
