# Supplementary material for: Trade-offs of lipid remodeling in a marine predator–prey interaction in response to phosphorus limitation
Source: Proc Natl Acad Sci U S A. 2022 Aug 29;119(36):e2203057119. doi: 10.1073/pnas.2203057119 (PMC9457565; doi:10.1073/pnas.2203057119)
Supplement: Supplementary File [file pnas.2203057119.sapp.pdf]

Supplementary Information

Supplementary Table S1: Representative studies stating the geographic distribution by isolation and molecular detection of *Uronema marinum* strains around the world

Sign	Environment	Isolation or detection by 18S rRNA	Reference
	New Zealand fish farm	Isolation	Anderson et al., 2009
	Antartica, Ross Sea	Isolation	Coppellotti et al., 1994
	China, fish farm	Isolation	Du et al., 2019
	Spain, Australia, Croatia, Norvegia, fish farm	Isolation and sequence	Dykova et.al, 2010
	USA (Georgia), sediment	Isolation	First et al., 2009
	Denmark, sediment	Isolation	Glud et al., 1999
	USA (New York), water	Isolation	Hanna et al., 1974
	South Korea, fish farm	Sequence	Harikrishnan et al., 2011
	India, water	Isolation	Kalavati et al., 1997
	USA (Massachusetts), water and sediment	Isolation	Kaneshiro et al., 1976
	South Korea, fish farm	Isolation	Kwon et al., 2003
	China, fish farm	Sequence	Li et. Al, 2018
	Artic, Antartic, water	Sequence	Nitsche et al., 2015
	UK, sediment	Isolation	Parker et al., 1976
	Mexico, fish farm	Isolation	Pérez-Bustamante et al., 2021
	Malaysia, Portugal, Greece, USA (Florida, California), water	Isolation	Pérez-Uz et., al 1995
	USA (Maine), fish farm	Isolation	Plunket et al., 1978
	South Korea, fish farm	Isolation	Song et al., 2009
	China, water	Isolation	Zheng et al., 2015
	South Korea, fish farm	Isolation	Whang et. al, 2013

Supplementary Table S2 Bacterial strains and plasmids used in this study

Strains or plasmids	Relevant characteristics	Source or Reference
Uronema marinum	Isolated from coastal seawater from Qingdao, China	Teng et al., 2021
Phaeobacter sp. MED193		
Wild-type (WT)	Isolated from Mediterranean Sea	
Δ*plcP*	Deletion mutant ΔMED193_17359	Sebastian et al., 2016
WT [pBBR-KanR-p(aphII)-sfGFP]	pBBR1MCS-2 carrying the coding sequence of sfGFP under the control of constitutive aphII promoter; formely named pBM44	Teng et al., 2021
WT [pBBR-GentR-p(aphII)-mCherry]	pBBR1MCS-5 carrying the coding sequence of mCherry under the control of constitutive aphII promoter; formely named pBM45	Teng et al., 2021
Δ*plcP* [pBBR-KanR-p(aphII)-mCherry]	pBBR1MCS-2 carrying the coding sequence of mchery under the control of constitutive aphII promoter	This study
Escherichia coli		
S17.1 lamda-pir		Lab stock
OP50		Lab stock
Plasmids		
pBBR-MCS		Kovach et al., 1995

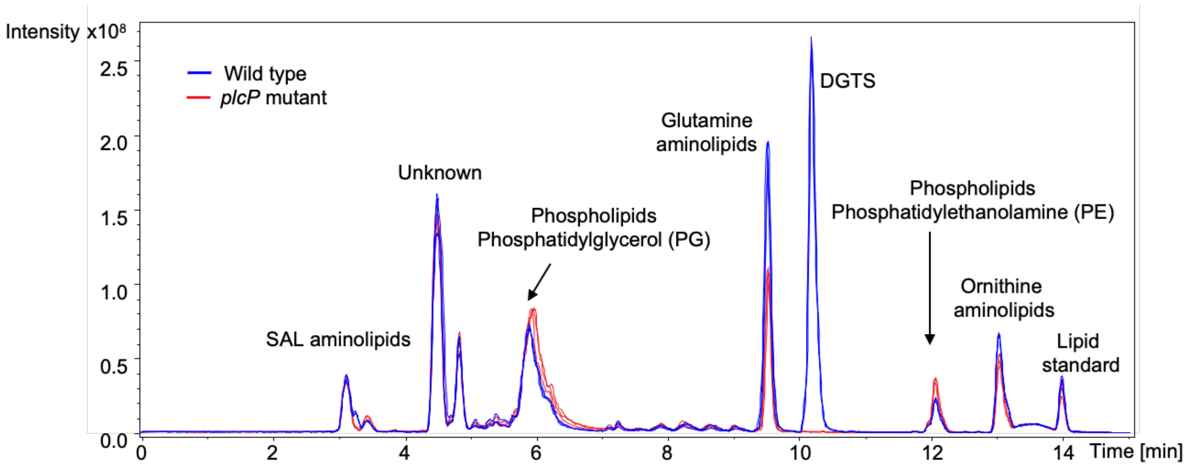

Supplementary Figure S1: LC-MS chromatogram showing the lipid profile of the wild type and the *plcP* mutant of *Phaeobacter* sp. MED193 cultivated in P-deplete artificial seawater. The *plcP* mutant is unable to produce the surrogate lipid diacylglyceryl trimethylhomoserine (DGTS) (red lines). SAL, sulfur-containing aminolipids.

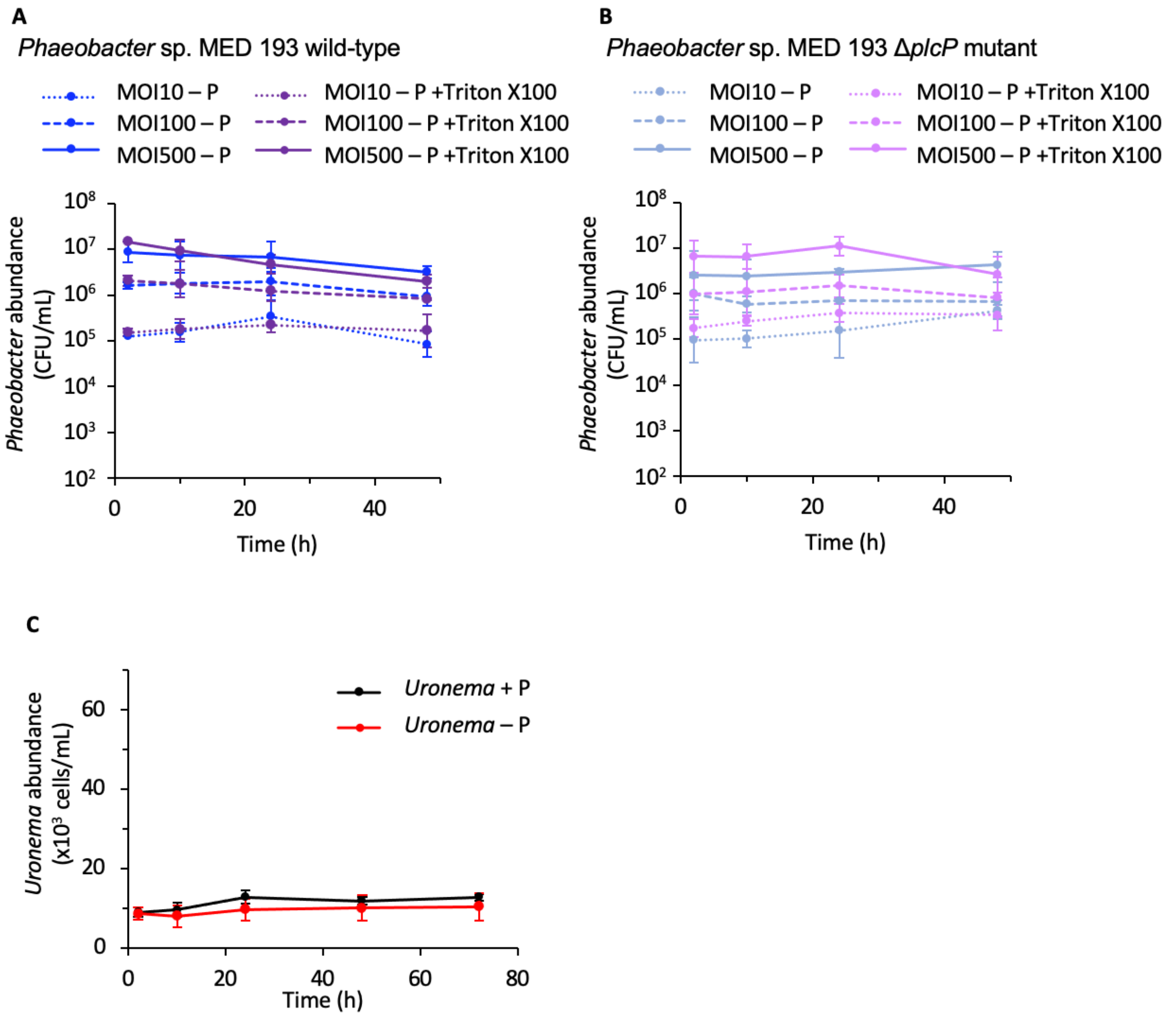

Supplementary Figure S2: Assessment of ciliate and bacterial growth in ASW medium and the effect of Triton X-100 on bacterial mortality. Growth of *Phaeobacter* sp. MED193 WT (A) and the Δ*plcP* mutant (B) in P-deplete ASW medium at a MOI 10 (dotted lines), MOI 100 (dashed lines) and MOI 500 (solid lines) before (purple) and after the addition of Triton-X100 (blue) over a 48h period. Growth of *Uronema marinum* (C) in P-replete ASW medium (black) and P-deplete ASW medium (red) over a 72h period. Values are the mean of three independent biological replicates, and error bars represent standard deviations.

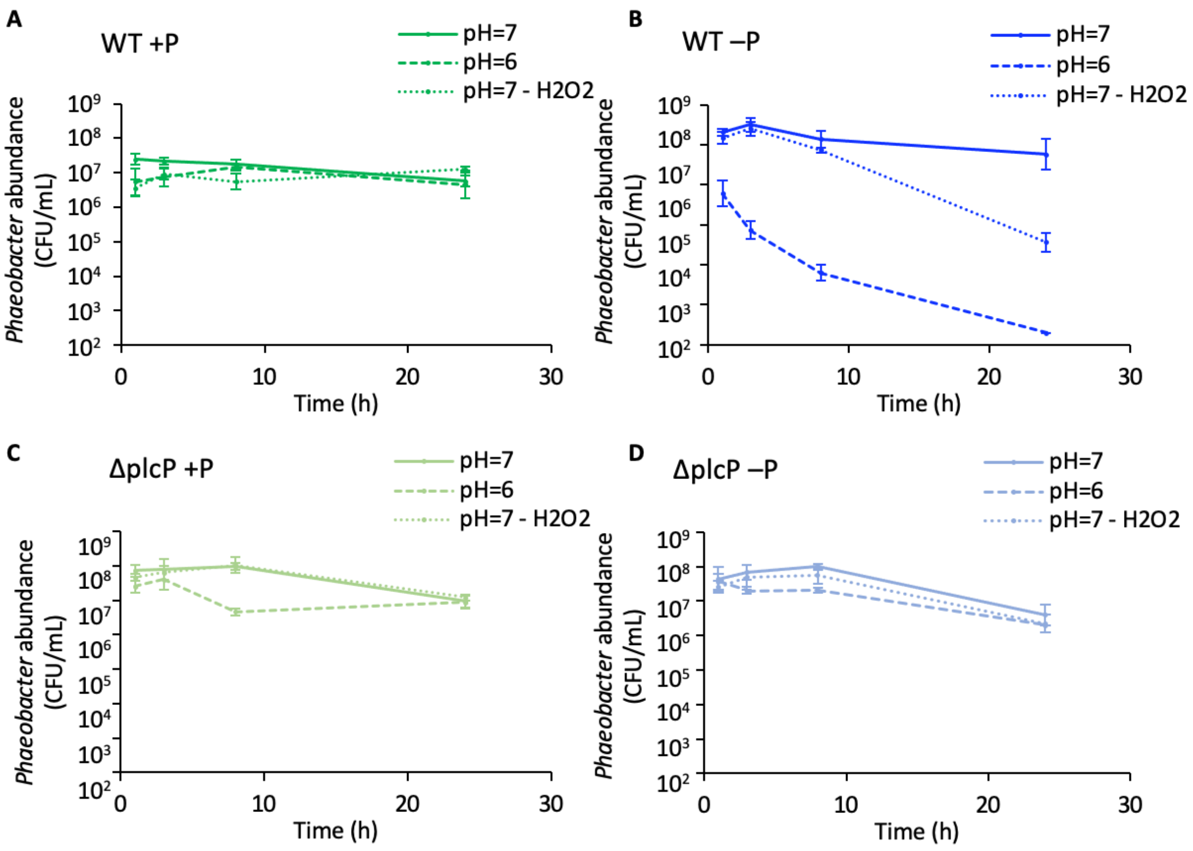

Supplementary Figure S3: Lipid remodeled *Phaeobacter* sp. MED193 is more sensitive to acidic pH and oxidative stress. Growth of *Phaeobacter* sp. MED193 WT (A and B) and the Δ*plcP* mutant (C and D) after growth in P-replete ASW (Green) or P-deplete ASW (Blue) followed by incubation in MES buffer at pH=7 (solid lines), pH=6 (dashed lines) and pH=7 with 70 µM H_2O_2 (dotted lines) over a 48h period.

References

S. A. Anderson, D. A. Hulston, S. M. McVeagh, V. L. Webb, P. J. Smith, In vitro culture and cryopreservation of *Uronema marinum* isolated from farmed New Zealand groper (*Polyprion oxygeneios*). *J Microbiol Methods* **79**, 62-66 (2009).

O. Coppellotti, Effects of cadmium on *Uronema marinum* (Ciliophora, Scuticociliatida) from Antarctica. Acta *Protozoologica* **33**, 159-167 (1994).

G. Du et al., Ciliate *Uronema marinum* is the causative agent of scuticociliatosis in farm raised turbot *Scophthalmus maximus*. *Journal of Oceanology and Limnology* **37**, 1726-1735 (2019).

I. Dykova, T. Tyml, M. Kostka, H. Peckova, Strains of *Uronema marinum* (Scuticociliatia) co-isolated with amoebae of the genus *Neoparamoeba*. *Dis Aquat Organ* **89**, 71-77 (2010).

M. R. First, J. T. Hollibaugh, The model high molecular weight DOC compound, dextran, is ingested by the benthic ciliate *Uronema marinum* but does not supplement ciliate growth. *Aquatic microbial ecology* **57**, 79-87 (2009).

R. N. Glud, T. Fenchel, The importance of ciliates for interstitial solute transport in benthic communities. *Marine Ecology Progress* **186**, 87-93 (1999).

B. A. Hanna, D. M. Lilly, Growth of *Uronema marinum* in chemically defined medium. *Marine biology* **26**, 153-160 (1974).

R. Harikrishnan, M. C. Kim, J. S. Kim, C. Balasundaram, M. S. Heo, Immunomodulatory effect of probiotics enriched diets on *Uronema marinum* infected olive flounder. *Fish Shellfish Immunol* **30**, 964-971 (2011).

C. Kalavati, A. V. Raman, J. Vaidehi, V. R. Bharathi, Effects of pollution on planktonic ciliates in Visakhapatnam harbour. (1997).

E. S. Kaneshiro, G. G. Holz, Jr., Observations on the ultrastructure of *Uronema* spp., marine scuticociliates. *J Protozool* **23**, 503-517 (1976).

S. R. Kwon, C. S. Kim, K. H. Kim, Differences between short-and long-term cultures of *Uronema marinum* (Ciliophora: Scuticociliatida) in chemiluminescence inhibitory activity, antioxidative enzyme and protease activity. *Aquaculture* **221**, 107-114 (2003).

R. Li et al., Mitochondrial genome sequencing and analysis of scuticociliates (*Uronema marinum*) isolated from *Takifugu rubripes*. *Mitochondrial DNA B Resour* **3**, 736-737 (2018).

F. Nitsche, H. Arndt, Comparison of similar Arctic and Antarctic morphotypes of heterotrophic protists regarding their genotypes and ecotypes. *Protist* **166**, 42-57 (2015).

J. G. Parker, Cultural characteristics of the marine ciliated protozoan, *Uronema marinum* Dujardin. *Journal of Experimental Marine Biology and Ecology* **24**, 213-226 (1976).

I. S. Perez-Bustamante, J. Caceres-Martinez, R. Cruz-Flores, R. Vasquez-Yeomans, First record of dark leathery surface of geoduck clams in *Panopea globosa*. *J Invertebr Pathol* **180**, 107543 (2021).

B. Pérez-Uz, Growth rate variability in geographically diverse clones of *Uronema* (Ciliophora: Scuticociliatida). *FEMS Microbiology ecology* **16**, 193-203 (1995).

L. Plunket, H. Hidu, The role of *Uronema marinum* (Protozoa) in oyster hatchery production. *Aquaculture* **15**, 219-225 (1978).

J. Y. Song et al., Pathogenicity of *Miamiensis avidus* (syn. *Philasterides dicentrarchi*), *Pseudocohnilembus persalinus*, *Pseudocohnilembus hargisi* and *Uronema marinum* (Ciliophora, Scuticociliatida). *Dis Aquat Organ* **83**, 133-143 (2009).

W. Zheng, F. Gao, A. Warren, High-density cultivation of the marine ciliate *Uronema marinum* (Ciliophora, Oligohymenophorea) in axenic medium. *Acta Protozool*. **54**, 325-330 (2015).

I. Whang, H. S. Kang, J. Lee, Identification of scuticociliates (*Pseudocohnilembus persalinus*, *P. longisetus*, *Uronema marinum* and *Miamiensis avidus*) based on the cox1 sequence. *Parasitol Int* **62**, 7-13 (2013).

Kovach, M. E., Elzer, P. H., Hill, D. S., Robertson, G. T., Farris, M. A., Roop II, R. M., & Peterson, K. M. (1995). Four new derivatives of the broad-host-range cloning vector pBBR1MCS, carrying different antibiotic-resistance cassettes. *Gene* **166**, 175-176.

Sebastián, M., Smith, A. F., González, J. M., Fredricks, H. F., Van Mooy, B., Koblížek, M., ... & Chen, Y. (2016). Lipid remodelling is a widespread strategy in marine heterotrophic bacteria upon phosphorus deficiency. *The ISME Journal* **10**, 968-978.

Teng, Z. J., Wang, P., Chen, X. L., Guillonneau, R., Li, C. Y., Zou, S. B., ... & Zhang, Y. Z. (2021). Acrylate protects a marine bacterium from grazing by a ciliate predator. *Nature Microbiology*, **6**, 1351-1356.
